# Cement Spacer versus Endoprosthesis as Reconstruction Modality after Limb Salvage for Shoulder Malignancies: Systematic Review and Meta-Analysis

**DOI:** 10.15388/Amed.2026.33.1.1

**Published:** 2026-07-22

**Authors:** Akshat Gupta, Divesh Jalan, Nishith Gupta, Nupur Aggarwal, Kshitish Behera, Princi Jain

**Affiliations:** 1 Department of Orthopaedics, All India Institute of Medical Sciences, Rajkot, Gujarat, India E-mail:; 2 Central Institute of Orthopaedics, Vardhman Mahavir Medical College & Safdarjung Hospital, New Delhi, India E-mail: ORCID ID; 3 Orthopaedic Oncology, Narayana Hospitals, Kolkata, West Bengal, India; 4 Department of Burns & Plastic Surgery, All India Institute of Medical Sciences, Rajkot, Gujarat, India; 5 Orthopaedic Oncology, Bagchi Sri Shankara Cancer Centre and Research Institute, Bhubaneshwar, Odisha, India E-mail:; 6 Department of Medicine, Lady Hardinge Medical College, New Delhi, India E-mail:

**Keywords:** shoulder, humerus, neoplasm, malignancy, cement spacer, arthroplasty, petis, žastikaulis, navikas, piktybinis navikas, cementinis tarpiklis, artroplastika

## Abstract

**Background:**

Limb salvage is the mainstay of surgical treatment for primary sarcomas as well as metastatic lesions of the proximal humerus. This involves wide excision of the involved humeral segment followed by a reconstruction of the defect. The purpose of this review was to determine which reconstruction modality offers a better overall functional outcome.

**Methods:**

After registering a review with the *PROSPERO* database (ID No. CRD 42025642215), *PubMed* and *Cochrane Library* databases were systematically screened for relevant studies meeting our inclusion criteria. The *Methodological Index for Non-randomized studies* (MINORs) questionnaire was the primary *Risk Of Bias* (ROB) assessment tool. The included articles were compared with respect to their intra-operative parameters, functional outcome scores, postoperative shoulder *Range Of Motion* (ROM), as well as complication rates.

**Results:**

A total of eight studies, involving 348 subjects in total, were included in this review, out of which, only four articles were found eligible for meta-analysis. Primary malignancy of the bone was the most common diagnosis (Osteogenic sarcoma followed by Ewing sarcoma). A higher frequency of axillary nerve sacrifice was seen in patients undergoing spacer implantation. *Musculoskeletal Tumour Society Scores* (MSTS) were used as the chief functional outcome determinant and were significantly better in patients undergoing arthroplasty (73.7 ± 7.9 versus 68.9 ± 8.5). A similar trend was observed with regards to shoulder flexion, extension and abduction. The most common complication noted with a cement spacer was proximal implant migration followed by implant failure. On the other hand, dislocation or subluxation of the prosthesis was the most frequently reported issue with arthroplasty. Overall, the local recurrence rate was 9.5%.

**Conclusion:**

Cement spacers are ideally suited for patients with a poor socio-economic background, aggressive lesions and a shorter life expectancy, while joint replacement is the reconstructive modality of choice in low-grade, well differentiated, isolated malignancies with negligible chances of sacrificing the shoulder abductor mechanism.

## Abstract (it)

**Įvadas:**

Išsaugoti galūnę yra pagrindinis pirminių sarkomų bei proksimalinio žastikaulio metastazinių pažeidimų chirurginio gydymo tikslas. Tam pirmiausia atliekamas platus pažeisto žastikaulio segmento išpjaustymas, paskui atliekama rekonstrukcija. Šia apžvalga siekta nustatyti, kuris rekonstrukcijos metodas užtikrina geresnius bendrus funkcinius rezultatus.

**Metodai:**

Užregistravus apžvalgą PROSPERO duomenų bazėje (ID Nr. CRD 42025642215), „PubMed“ ir „Cochrane Library“ duomenų bazėse buvo sistemingai ieškoma tyrimų, atitinkančių mūsų atrankos kriterijus. Metodologinis neatsitiktinių tyrimų indeksas (MINORs) klausimynas buvo pagrindinė šališkumo rizikos (ROB) vertinimo priemonė. Įtraukti straipsniai lyginti pagal jų intraoperacinius parametrus, funkcinių rezultatų balus, pooperacinę peties judesių amplitudę (ROM), taip pat komplikacijų dažnį.

**Rezultatai:**

Į šią apžvalgą įtraukti aštuoni tyrimai su 348 tiriamaisiais, iš kurių tik keturi straipsniai buvo pripažinti tinkami metaanalizei. Dažniausia diagnozė buvo pirminis kaulų piktybinis navikas (osteogeninė sarkoma, paskui Ewingo sarkoma). Pacientų, kuriems buvo implantuojamas tarpinis implantas, dažniau buvo aukojamas pažastinis nervas. Pagrindinis funkcinių rezultatų rodiklis buvo „Musculoskeletal Tumour Society“ balai (MSTS), jie buvo kur kas geresni pacientų, kuriems buvo atlikta artroplastika (73,7 + 7,9, palyginti su 68,9 + 8,5). Panaši tendencija buvo nustatyta ir peties lenkimo, tiesimo bei atitraukimo atžvilgiu. Dažniausia komplikacija, susijusi su cementiniu tarpikliu, buvo proksimalinė implanto migracija, o paskui implanto gedimas. Kita vertus, protezo išnirimas ar subliuksacija buvo dažniausia įvardijama problema, susijusi su artroplastika. Bendras vietinio recidyvo dažnis buvo 9,5 procento.

**Išvada:**

Cementinis tarpiklis yra laikinas sąnario implantas, naudojamas sąnario endoprotezavimo operacijose, idealiai tinka pacientams, kurių žema socialinė ir ekonominė padėtis, agresyvūs navikai ir trumpesnė gyvenimo trukmė, o sąnario pakeitimas yra tinkamiausias rekonstrukcinis metodas esant ankstyvos stadijos, gerai diferencijuotiems, izoliuotiems piktybiniams navikams, kai peties atitraukimo (abdukcijos) mechanizmo praradimo tikimybė yra nereikšminga.

## Introduction

The proximal humerus is one of the most common sites for primary and secondary bony malignancies. The estimated incidence rates of *Osteogenic Sarcoma* (OGS) and *Ewing Sarcoma* (EWS) involving the proximal humerus are 10–15% and 10%, respectively. [1,2] It is also the second most common location for metastatic involvement and the fourth most common site afflicted by soft tissue sarcomas (STS). [1,3,4]

While radical excision in the form of amputation was the mainstay of surgical treatment in the past, the trend has now shifted towards limb salvage. [5-7] The latter offers functionally and cosmetically better outcomes while offering an acceptable oncological outcome. Concerning post-surgical resection, several reconstructive options have been described in the literature, such as endoprosthesis, *Cement Spacer* (CS), *Osteoarticular Allografts* (OAA), *Allograft-Prosthetic Composite* (APC), Clavicula Pro Humero, *Free Fibula* (FF) transfers, etc. [7-12] Each technique involves specific pros and cons. The biological reconstruction options can be complicated by fracture, infection, donor site morbidity, as well as bony and soft tissue healing issues due to ongoing chemotherapy and radiotherapy. [9,10,13] On the other hand, the Endoprostheses are denoted by higher chances of dislocation and are limited by their cost-effectiveness. [14-16]

In the above-outlined context, CS has emerged as a low-cost alternative with equivalent functional outcomes and relatively favourable survival rates in patients with proximal humerus malignancies. [6,14-17] Systematic reviews on the same have shown that while arthroplasty is a viable option in patients with a longer life expectancy and a preserved deltoid and/or axillary nerve, spacers are more useful in cases with a non-functioning abductor mechanism, multiple aggressive lesions, and a poor socio-economic background. [13,18] However, a detailed statistical comparison between the above-outlined two techniques has not yet been described. Moreover, several research articles have been published on this topic in recent years. [14,15,19] In light of these observations, we conceptualized this study with the following objectives: (i) a review of available literature to assess the functional outcomes following proximal humerus resection and reconstruction using Nail/Plate cement spacers (NCS/PCS), and (ii) an objective comparison of NCS/PCS with endoprosthesis in terms of the shoulder functionality and outcome.

## Materials and Methods

No ethical waiver was needed for this study as it was a systematic review of available literature.

### Protocol registration

The study protocol was registered with *PROSPERO* (ID number: CRD 42025642215).

### Inclusion and Exclusion Criteria

The population, intervention, control, and outcome (PICO) format was used to formulate the research question and define inclusion and exclusion criteria for the review. All research articles included the following categories of patients: (i) suffering from histopathologically proven primary or secondary malignancy of the proximal humerus; (ii) those who underwent excision of the lesion and reconstruction of the skeletal defect with NCS/PCS (Intervention) and endoprosthesis (Control); (iii) who had an average follow-up of at least two years, and (iv) who underwent outcome assessment by using specifically defined functional evaluation scores. These four categories were included in our research sample. Single-arm studies, conference abstracts, isolated case reports, and review articles were excluded from the analysis.

### Search strategy

A systematic search of the *PubMed*/*MEDLINE* and *Cochrane Library* databases was carried out by two independent reviewers (AG and NG), employing a search strategy that was conceptualized *a priori*. Additionally, results from *Google Scholar* and references of full-text articles obtained after primary search were screened, and the relevant articles were incorporated into the review. The following *Medical Subject Headings* (MeSH) were used to refine our search – ‘tumour’, ‘tumor’, ‘cancer’, ‘sarcoma’, ‘neoplasm’, ‘neoplastic’, ‘metastases’, ‘metastasis’, ‘secondaries’, ‘bone’, ‘bony’, ‘proximal humerus’, ‘cement spacer’, ‘arthroplasty’, ‘joint replacement’, ‘endoprosthesis’, ‘endoprosthetic’ and ‘megaprosthesis’. These were then combined by using specific limiters and Boolean operatives to yield results. The time frame for the literature review was set at inception till the completion of this study. No additional filters were added. Our detailed search strategy is described in Appendix A.

### Study selection

Literature was screened as per the *Preferred Reporting Items of Systematic Reviews and Meta-Analyses* (PRISMA) guidelines. [20] In the first step, titles and abstracts of the primary search results were assessed. Irrelevant as well as duplicate citations were excluded, and the remainder were carried forward to the next stage of the screening process. This involved retrieving full-length texts of the articles being reviewed and matching them with the laid-down inclusion/exclusion criteria. Discrepancies between the reviewers were settled by further discussion, and, in case of an impasse, a third reviewer (KB) was consulted.

### Risk-of-bias (ROB) assessment

Two independent reviewers (AG and NA) evaluated the included studies for methodological strength and quality. The tool used for the latter was the *Methodological Index for Non-Randomized Studies* (MINORS) questionnaire (Appendix B). [21] Discrepancies during ROB assessment were resolved through mutual consensus, and, when required, a senior reviewer (DJ) made the final decision.

Both comparative and non-comparative studies were analyzed and graded as ‘Good’, ‘Fair’, and ‘Poor’, based on their respective scores (Appendix C).

### Outcome measures and scores

The primary functional outcome evaluation parameters used in this review were the *Musculoskeletal Tumour Society* (MSTS) scores [22], the shoulder range of motion (ROM), and the frequency as well as the nature of associated surgical complications.

Local and Distant recurrence (LR and DR) rates were denoted as the chief oncological outcome determinants.

### Data analysis

The selected studies were first imported to a reference manager software (*Zotero*, version 6.0.30). The demographic, operative, and functional evaluation scores of individual studies were then summarized on a *Microsoft Excel* Spreadsheet (*Office 365*; Microsoft Corporation, Redmond, WA). This included the author/year of publication, the sample size, the mean age of the study population, the sex ratio, the average follow-up, histopathological diagnosis, the number of cases undergoing axillary nerve and deltoid resection, the average resection length (RL), the status of margins, ROM, MSTS scores, and the complication rates and frequency of LR as well as DR. All quantitative data were then expressed as mean ± standard deviation (SD).

For Meta-analysis, the *RevMan* 5.3© software designed by the *Cochrane* collaboration was used.^23^ Summaries of the intervention effects of each particular study were then pooled on a random effects model, and the odds ratio (Dichotomous outcomes), as well as the mean difference (Continuous outcomes) were calculated. The comparisons made included the following points: (i) axillary nerve resection, (ii) RL, (iii) Shoulder ROM, (iv) MSTS scores, and (v) the complication rates. Heterogeneity was estimated by using the I^2^ test. Significance was set at a *p*-value of <0.05 with 95% confidence interval (CI). Forest plots were used to graphically denote the comparison between the two techniques for each outcome of interest.

## Results

### Literature search

A total of 22 records were identified in the initial phase of the screening process. These included 19 articles from *PubMed* and three more after going through the references of the primary search results. No study meeting our inclusion criteria was found in the *Cochrane Library* database. Out of 22, only 10 studies were brought to the second stage of literature search. The rest were excluded on relevance, study design, etc.

These ten articles were then closely scrutinized by going through their full-length texts, and two more studies were excluded as the reconstruction strategy employed by the authors did not meet our review criteria.

Eight articles were finally included in our review, [6,7,14-17,19,24] of which, four were further meta-analysed. [7,14-16]

**Fig. 1:**
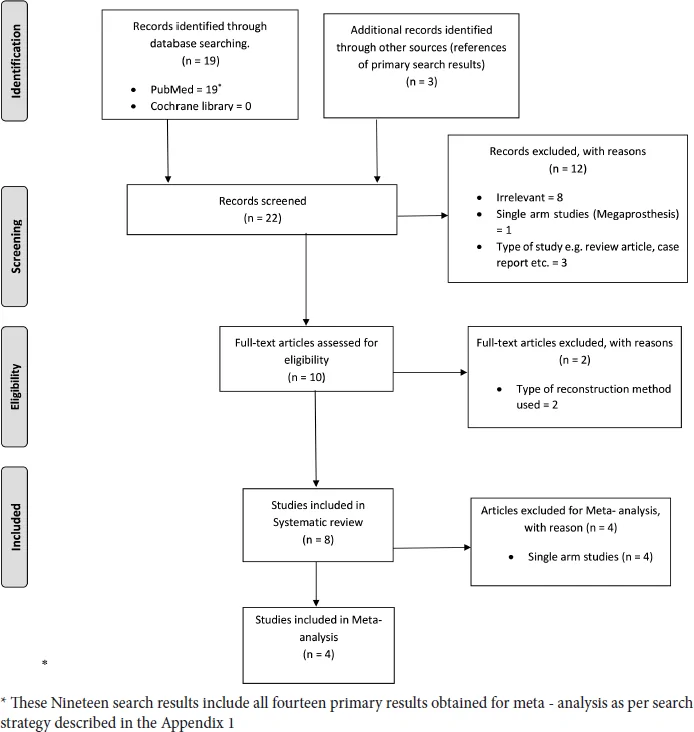
Flowchart depicting the screening process and study selection

The *PRISMA* flowchart depicting the sequence of literature searches is described in Figure 1.

### ROB assessment

Out of eight, only the studies by Manfrini et al. [7], Rafalla and Abdullah [16], Ebeid et al. [15], and Sehrawat et al. 14] were comparative double-arm studies.

Most articles (six out of eight or 75%) scored ‘Fair’ on the MINORS questionnaire. [6,7,14,16,17,19] One study was graded as ‘Good’ as it was a prospective analysis of retrospective data. [15]

While Guo et al. [24] scored only 7/16 and was denoted as ‘Poor’, it was included in the analysis as the study had focused exclusively on proximal humeral metastases and excluded patients with an estimated survival time of more than twelve months.

A summary of the ROB assessment of included articles is described in Appendix C.

### Population characteristics

A total of 348 subjects (eight studies) were included in this review. This included 276 patients who underwent proximal humerus reconstruction using CS (eight studies), while the remaining 72 (four studies) had an endoprosthesis placement. There were 177 males and 134 females (seven studies analysed) with an average age of 29.23±14.2 years.

The most common histopathological diagnosis was OGS (167/372, or 44.9%), followed by EWS (90/372, or 24.2%) and Chondrosarcoma (CS) (35/372, or 9.4%). Giant cell tumour (GCT) and secondary malignancies accounted for 30 (8.1%) and 29 (7.8%) cases, respectively. A detailed diagnostic distribution is given in Table 1.

**Table 1:** Baseline demographic characteristics of the included studies]

SNO.	STUDY	SAMPLE SIZE		MEAN AGE (years)	SEX RATIO (M: F)	DIAGNOSIS	AVERAGE FOLLOW-UP (years)
		CS	P				
1.	Manfrini et al (2011)	12	25	9.3 ± 2.1	NS	OGS – 50; EWS –11^*^	11.8 ± 8.4
2.	Kundu et al (2013)	14	NA	28.9 ± 12.9	6:8	OGS – 8; CS – 4; Met - 2	2.5 ± 1
3.	Rafalla and Abdullah (2017)	12	8	40.4 ± 12	6:14	OGS – 6; CS – 2; Met – 4; GCT – 2; CB – 2; Others – 4 (Myeloma – 2; Lymphoma – 2)	2.2 ± 1.3
4.	Guo et al (2019)	15	NA	56.3 ± 8.6	8:7	Met – 15	NA^¶^
5.	Gulia et al (2021)	142	NA	17.5	96:46	OGS – 64; 2 -11; 3 – 48; STS – 4; GCT – 14; 7 -1	2.8 ± 3.6
6.	Ebeid et al (2022)	39	19	27.5 ± 15.3	35:23	OGS – 22; CS – 12; EWS – 13; Met – 1; STS – 2; GCT – 5; CB – 2; Others – 1 (Lymphoma)	4.5 ± 3.7
7.	Sehrawat et al (2024)	20	20	24.9 + 10.2	18:22	OGS – 15; 2 – 5; 3 – 11; Met – 1; STS – 1; GCT -6; Others – 1 (Multiple myeloma)	2.6 ± 1
8.	Khan et al (2024)	22	NA	29 ± 17	8:14	OGS – 2; CS – 1; EWS – 7; Met – 6; GCT – 3; CB -2; Others – 1 (Multiple myeloma)	3.1 ± 1.5

CS – Cement Spacer; P – Prosthesis; OGS – Osteogenic Sarcoma; CS – Chondrosarcoma; EWS – Ewing Sarcoma; Met – Metastatic lesions; STS – Soft tissue sarcoma; GCT – Giant Cell tumour; CB – Chondroblastoma; NS – Not specified; NA – Not applicable * Total sample size of the study was 61 out of which 25 patients underwent arthroplasty and 12 had spacer reconstruction. However, the remaining 24 subjects were also analysed in order to calculate final diagnostic distribution. ¶ Subjects with survival time < 3 months and > 12 months were excluded from the study by authors

The average duration of the follow-up stood at 4.2±3.4 years (seven studies analysed in total).

### Operative parameters

The margin status was wide in 155 study subjects, marginal/intralesional in 13, and radical in the remaining seven (with five studies evaluated).

**Fig. 2:**
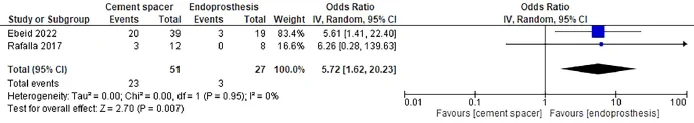
Forest plot depicting variation in the frequency of axillary nerve resection for either technique

A total of 196 patients underwent axillary nerve resection. Only two studies [15,16] directly compared the reconstruction techniques vis-à-vis the status of the axillary nerve. Quantitative analysis for the same aspect revealed a statistically significant difference (OR 5.72 [95% CI, 1.62, 20.23]) in favour of arthroplasty (Fig. 2).

**Fig. 3:**
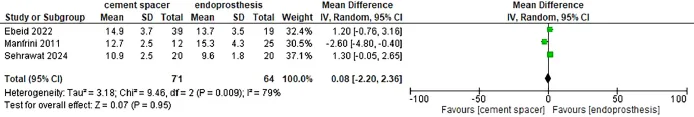
Forest plot depicting the comparison in resection length (RL) between the two procedures

Deltoid resection (complete/partial) was observed in 214 cases. The average resection length was 12.8±1.5 cm and 12.9±2.9 cm in the CS and prosthesis groups, respectively (MD 0.08[95% CI, -2.2,2.36]) (Fig. 3).

These observations are briefly summed up in Table 2.

**Table 2:** A brief summary of the intra-operative data of the included studies]

SNO.	STUDY	MARGINS	AXILLARY NERVE RESECTION		DELTOID RESECTION		MEAN RESECTION LENGTH (cm)	
			CS	P	CS	P	CS	P
1.	Manfrini et al (2011)	Wide – 43; Marginal – 11; Radical – 7	NS	NS	NS	NS	12.7 ± 2.5	15.3 ± 4.3
2.	Kundu et al (2013)	Wide – 14	14	NA	14	NA	12 ± 1.5	NA
3.	Rafalla and Abdullah (2017)	Wide – 20	3	0	NS	NS	NS	NS
4.	Guo et al (2019)	NS	NS	NA	NS	NA	NS	NA
5.	Gulia et al (2021)	NS	142	NA	142	NA	NS	NA
6.	Ebeid et al (2022)	Wide – 56; Marginal – 2	20	3	28	13	14.9 ± 3.7	13.7 ± 3.5
7.	Sehrawat et al (2024)	NS	NS	NS	NS	NS	10.9 ± 2.5	9.6 ± 1.8
8.	Khan et al (2024)	Wide – 22	14	NA	17	NA	13.3 ± 2.6	NA

CS – Cement Spacer; P – Prosthesis; NS – Not specified; NA – Not applicable

### Functional and oncological outcomes

**Fig. 4:**
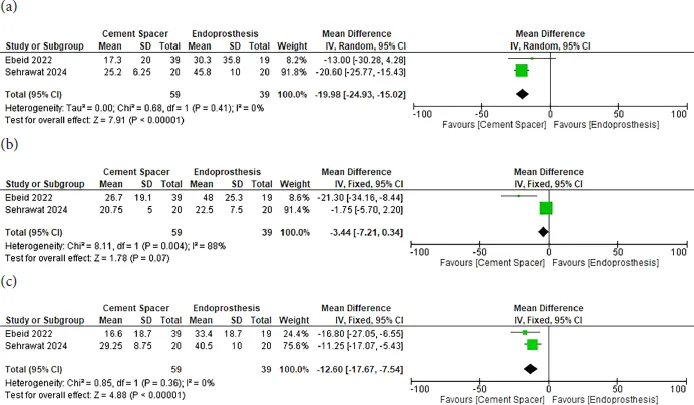
Forest plot depicting a comparison in the shoulder range of motion (ROM) in (a) Flexion, (b) Extension, and (c) Abduction, between the two reconstruction modalities

A direct comparison of the residual postoperative shoulder ROM in flexion, extension as well as abduction did not favour CS over endoprosthesis (MD -19.98[95% CI, -24.93, -15.02]) (Fig. 4a), (MD -3.44[95% CI, -7.21, 0.34]) (Fig. 4b), and (MD -12.6[95% CI, -17.67, -7.54]) (Fig. 4c).

**Fig. 5:**
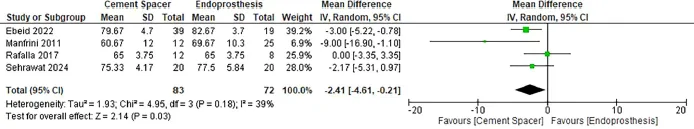
Forest plot depicting the Musculoskeletal Tumour Society Scores (MSTS) for either technique

The average MSTS score was also significantly higher in the patients undergoing arthroplasty (73.7 ± 7.9), when compared to the spacer group (68.9±8.5) (MD -2.41[95% CI, -4.61, -0.21]) (Fig. 5).

There were 64 complications (eight studies evaluated) in the CS cohort, of which, proximal migration of NCS/PCS was the most frequent problem encountered (24/64, or 37.5%), followed by implant failure (18.8%) and deep tissue infection (15.6%). On the other hand, subluxation or dislocation of the prosthesis was the most common complication (11/33, or 33.3%) in patients undergoing joint replacement. Osteolysis without implant loosening was observed in 8/33 (24.2%) subjects. Deep infection and prosthesis breakage/loosening were less commonly seen vis-à-vis the CS group.

**Fig. 6:**
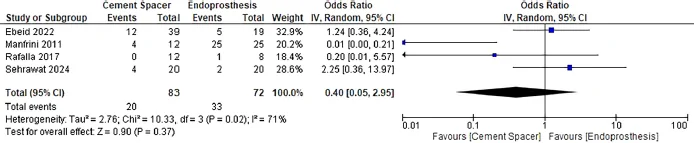
Forest plot depicting a comparison in complication rates between a cement spacer and endoprosthesis

Four studies [7,14-16] directly comparing the complication rates of the two techniques demonstrated higher incidents in the arthroplasty group (33) than in patients undergoing CS reconstruction (20). This difference, however, was not statistically significant (OR 0.40[95% CI,0.05,2.95]) (Fig. 6).

Oncological outcome assessment revealed 12 cases of LR and 26 cases of DR (all included studies were analysed in this regard).

A summary of the comparison of the outcomes of the two surgical techniques is given in Tables 3 and 4.

**Table 3:** A tabular comparison of the two techniques vis -a-vis their functional and oncological outcomes]

SNO.	STUDY	ROM		MSTS		RECURRENCE
		CS	P	CS	P	
1.	Manfrini et al (2011)	NS	NS	60.67 ± 12	69.67 ± 10.3	LR -5
2.	Kundu et al (2013)	NS	NA	63.64 ± 8	NA	LR – 1; DR - 3
3.	Rafalla and Abdullah (2017)	NS	NS	65 ± 3.8	65 ± 3.8	LR – 1; DR - 3
4.	Guo et al (2019)	NS	NA	57.1 ± 9.9	NA	DR - 12
5.	Gulia et al (2021)	NS	NA	71 ± 5	NA	NS
6.	Ebeid et al (2022)	F – 17.3 ± 20; E – 26.7 ± 19.1; Ab -16.6 ± 18.7	F – 30.3 ± 35.8; E – 48 ± 25.3; Ab – 33.4 ± 18.7	79.67 ± 4.7	82.67 ± 3.7	LR – 6; DR - 8
7.	Sehrawat et al (2024)	F – 25.2 ± 6.25; E – 20.75 ± 5; Ab – 29.25 ± 8.75	F – 45.8 ± 10; E – 22.5 ± 7.5; Ab – 40.5 ± 10	75.3 ± 4.17	77.5 ± 5.8	LR - 3
8.	Khan et al (2024)	NS	NA	78.7 ± 9.7	NA	NS

CS – Cement Spacer; P – Prosthesis; NS – Not specified; NA – Not applicable; ROM – Range of motion; F – Shoulder Flexion; E – Shoulder Extension; Ab – Shoulder Abduction; MSTS – Musculoskeletal Tumour Society; LR – Local recurrence; DR – Distal recurrence

**Table 4:** Complications seen following proximal humerus reconstruction using either cement spacer or endoprosthesis]

SNO.	STUDY	RNP		Infection		Wound breakdown		Implant failure (loosening/breakage)		Proximal Migration		Subluxation/Dislocation		Others	
		CS	P	CS	P	CS	P	CS	P	CS	P	CS	P	CS	P
1.	Manfrini et al (2011)	0	1	1	3	0	0	1	3	0	0	0	9	2^#^	9^#^
2.	Kundu et al (2013)	1	NA	0	NA	0	NA	0	NA	0	NA	1	NA	0	NA
3.	Rafalla and Abdullah (2017)	0	0	0	0	0	0	0	0	0	0	0	1	0	0
4.	Guo et al (2019)	0	NA	0	NA	0	NA	0	NA	0	NA	0	NA	0	NA
5.	Gulia et al (2021)	0	NA	5	NA	3	NA	7	NA	22	NA	1	NA	2^$^	NA
6.	Ebeid et al (2022)	3	0	1	1	2	0	2	1	2	2	2	1	0	0
7.	Sehrawat et al (2024)	1	1	2	1	0	0	1	0	0	0	0	0	0	0
8.	Khan et al (2024)	0	NA	1	NA	0	NA	1	NA	0	NA	0	NA	0	NA

CS – Cement Spacer; P – Prosthesis; RNP – Radial Nerve Palsy; NA – Not applicable # - Included two cases of proximal osteolysis without loosening in the cement spacer group and eight cases of cortical resorption without loosening along with one patient of periprosthetic ossification in the endoprosthesis group $ Two patients had prominent acromion process (one was manged with partial excision and the other conservatively)

## Discussion

Prior to the late 1970s, most high-grade malignancies of the shoulder were treated with a forequarter amputation in order to attain oncologically safe surgical margins. All this changed, however, with Marcove et al. [25] who was the first to describe an *en-bloc* humeral inter scapulothoracic resection technique for such lesions. The authors demonstrated equivalent survival rates and local tumour control if compared with amputation. Over the years, advancements in cancer management strategies, such as radiation and chemotherapy regimens, surgical techniques, prosthesis designs, and better rehabilitation protocols, have led to limb salvage becoming the gold standard of surgical care for proximal humeral malignancies. About 80% of such neoplasms are nowadays successfully treated with a wide excision of the lesion followed by a reconstruction of the shoulder girdle. [26] However, the choice of ideal reconstruction modality is still a matter of debate. A lot depends on the patient’s age and activity levels, the extent of the tumour, the response to chemotherapy, the tumour characteristics, the requirement for postoperative radiotherapy, etc. [8,14] Most biological options have slightly better functionality due to the preservation and attachment of rotator cuff muscles to the soft tissue covering of the allograft. [8-10,12,13] As a result, we did not include it in our analysis and instead focused on comparing the outcomes between CS and endoprosthesis.

The most common malignancy seen in our study was OGS, followed by EWS. A wide excision of these lesions often requires resection of the shoulder abductor mechanism, the rotator cuff, and also axillary nerve sacrifice. While this restores the arm length and allows for satisfactory elbow and hand movements, the overall functional outcome of the shoulder is severely impacted. [6,13-15,17,18] Rafalla and Abdullah, in an analysis of 20 patients, proposed reserving CS for such patients and contraindicated RSA/megaprosthesis in cases with the following: (i) deficient glenoid bone stock; (ii) deltoid dysfunction; and (iii) poor socio-economic conditions. [16] The last point is particularly significant as, in our country (India), the approximate cost of a proximal humerus megaprosthesis is 600 USD compared to 80 USD for NCS/PCS. [6] Other disadvantages associated with the endoprosthesis use, particularly in children, include difficulty in implantation due to a narrow medullary canal and progressive cortical resorption at the implant-bone interface. [7] Loss of the soft tissue cover around the shoulder secondary to humeral excision may also cause the implanted spacer or prosthesis to subluxate/dislocate. [14,27,28] To obviate this shortcoming, Wang et al. recommended using a propylene mesh to augment the joint capsule and stabilize the reconstruction. [27] This was upheld by Sehrawat et al. [14] and Khan et al. [19] who used meshplasty anchored to the glenoid/capsular remnants and passed through the endoprosthesis/spacer. Reattachment of the remaining muscles, e.g., deltoid to the short biceps or pectoralis major, short biceps to the long biceps, and the rotator cuff to the implant, with heavy non-absorbable sutures such as Ethibond Excel© (Ethicon division, Johnson and Johnson) has also been shown to improve long term functional outcomes following proximal humerus reconstruction. [15,17]

The most commonly used tool by most authors to assess the functional outcomes was the MSTS score. This is a physician-reported instrument; a numerical score of 0–5 is assigned for each of the following six categories for upper extremity evaluation, notably, pain, functional activity, emotional acceptance, hand positioning, dexterity, and lifting ability, and then, the output is converted to a percentage scale. [29] We reported an average MSTS score of 68.8±8.5 for the CS group of patients. Similar findings were noted by Aiba et al. [18] in a review describing the various reconstructive options after Type I and V Malawer resection of the shoulder girdle. In comparison, patients undergoing endoprosthetic replacement of the proximal humerus had significantly higher scores (73.7±7.9) and active flexion, extension, and abduction ROM. In a systematic analysis of 141 patients from 10 studies, Teunis et al. [30] reported MSTS scores ranging from 61% to 77% with arthroplasty. Another review comparing endoprosthesis with CS for shoulder reconstruction following a wide excision of metastatic lesions showed significantly better functional outcomes with joint replacement (MSTS 73.2%, range 64–87.5%) vis-à-vis spacer (MSTS 69.1%, range: 57–81.65%). [13] Sehrawat et al. [14] used the *Disabilities of Arm, Shoulder and Hand* (DASH) questionnaire, shoulder ROM, and the MSTS scores to compare the two reconstruction techniques objectively. They reported better outcomes with endoprosthesis in all domains. Ebeid et al. [15] attributed this higher functionality, as well as an increased ROM, to selection bias. The authors remarked that, as most patients undergoing arthroplasty had a preserved shoulder abductor mechanism and an intact axillary nerve, the functional outcomes reported in them were likely to be better. As a result, patients belonging to each reconstruction modality were divided into two groups – with and without axillary nerve sacrifice to eliminate confounding – and then compared again. No statistically significant difference in the functional outcome was observed this time. In our opinion, despite the unique biomechanical design of RSA and megaprostheses, any significant difference observed between the two reconstruction modalities (spacer and arthroplasty) has more to do with the availability of an intact abductor mechanism as well as a functioning axillary nerve around the shoulder joint, rather than the prosthesis design itself.

We observed a higher postoperative complication rate with endoprosthesis than CS, a finding also reported in previous reviews. [13,18] The overall incidence of complications following proximal humerus resection and arthroplasty reconstruction is 20–45%. [31-33] The most frequent issue is prosthesis dislocation/subluxation, followed by infection and aseptic loosening. [5,18] However, some authors have reported only a few cases of prosthesis instability. [14-16] The recommended treatment for prosthesis dislocation is closed reduction followed by a period of immobilization. In case of recurrence or delayed presentation, revision surgery with a thicker polyethylene insert with/without glenoid revision can be considered. [34] Many authors have also recommended using a synthetic/biological mesh during the reconstruction anchored to the glenoidal labrum in order to mitigate the risk of dislocation. [14,17,35,36] Management of prosthetic joint infection involves irrigation with debridement of the surgical bed in patients with an early onset infection, with one- or two-stage exchange arthroplasty recommended in chronic cases. [5] Other reported complications include proximal migration of the implant, radial nerve injury, implant failure, wound-related complications, osteolysis at the implant-bone interface, etc. [8,13-18] In our review, the incidence of proximal implant migration in the spacer group was relatively high (24/64, or 37.5%). While migration can also be seen following arthroplasty, the need for revision is much greater with CS owing to the sharp end of the incorporated nail/plate, which causes significant discomfort to the patient. Gulia et al. [6] opined that adding a blob of cement at the proximal end of the spacer effectively reduces the frequency of proximal implant migration along with related complications. Overall, the LR observed in our study was 9.46%, which is comparable to the 5–11% rate described in the literature. [14,15,37,38]

This review was written despite the presence of a similar study by ARMA Helmy et al. [13] and Aiba et al. [18] about a year ago. We produced this paper since: (i) we aimed to compare the two reconstruction techniques more comprehensively, viz. spacer versus arthroplasty. Hence, we included patients afflicted by both primary and secondary malignancies of the proximal humerus. This was in stark contrast to ARMA Helmy et al. [13] who limited their study exclusively to metastatic lesions of the proximal humerus; also, (ii) a few studies [14,19] have been added to the literature only recently, and they did not find mention in either of the previous reviews; and, finally, (iii) ours is the only study so far which has performed a meta-analysis on the topic and, thus, can give a more objective statement on the reliability of CS as a reconstructive modality following wide resection of the proximal humerus.

Nevertheless, our study involves a few limitations. First, only one study out of eight was prospective in design. A meta-analysis of retrospective articles is undesirable, given the propensity for confounding and selection bias. However, this was the only feasible option due to insufficient randomized control trials (RCTs) or good-quality prospective comparative studies. Secondly, prostheses used by different authors may vary in design, modularity, and composition. Comparing individual implant designs and megaprosthesis with RSA is beyond the scope of this review. Finally, variations in the study quality, patient characteristics, the choice of implant, and rehabilitation protocols resulted in considerable heterogeneity when the studies were analysed. All data entries were double-checked to mitigate this, and a random effects model was employed during quantitative analysis.

Despite these shortcomings, all precautions were taken to ensure the methodological robustness of this review, such as: (i) using the PICO format to formulate the research question; (ii) reviewing multiple databases by PRISMA guidelines; (iii) minimizing bias by having independent reviewers screen search results and perform ROB assessment; and (iv) having a third expert to resolve any discrepancies encountered above.

## Conclusion

Cement spacers offer a functionally robust and cost-effective solution following proximal humerus resection in primary/secondary shoulder malignancies. They are suited for cases involving a non-functional deltoid and/or axillary nerve, multiple metastatic lesions and a shorter life expectancy. When compared with endoprostheses, however, no significant difference was noted in terms of functional or oncological outcomes.

## Appendix A. Detailed search strategy including terms and Boolean operators for electronic database search

A)PubMed
  1. Sarcoma (210,198)
  2. Tumour (5,046,197)
  3. Tumor (5,046,197)
  4. Cancer (5,272,799)
  5. Neoplasm (4,160,542)
  6. Neoplastic (370,715)
  7. Secondaries (1,443,397)
  8. Metastasis (487,966)
  9. Metastases (439,981)
  10. (1) OR (2) OR (3) OR (4) OR (5) OR (6) OR (7) OR (8) OR (9) (6,833,965)
  11. Bone (1,558,168)
  12. Bony (53,933)
  13. (11) OR (12) (1,576,902)
  14. Proximal humerus (7,134)
  15. Cement spacer (1,378)
  16. Joint replacement (98,219)
  17. Megaprosthesis (450)
  18. Endoprosthesis (620,301)
  19. Endoprosthetic (1,767)
  20. Arthroplasty (132,507)
  21. (16) OR (17) OR (18) OR (19) OR (20) (719,490)
  22. (10) AND (13) AND (14) AND (15) (19)
  23. (22) AND (21) (14)
B)Cochrane Library Database:
  1. (Sarcoma): ti, ab, kw (3,174)
  2. (Tumour): ti, ab, kw (89,142)
  3. (Tumor): ti, ab, kw (89,142)
  4. (Cancer): ti, ab, kw (243,251)
  5. (Neoplasm): ti, ab, kw (39,465)
  6. (Neoplastic): ti, ab, kw (3,499)
  7. (Secondaries): ti, ab, kw (52)
  8. (Metastasis): ti, ab, kw (28,206)
  9. (Metastases): ti, ab, kw (14,213)
  10. 1 OR 2 OR 3 OR 4 OR 5 OR 6 OR 7 OR 8 OR 9 (279,095)
  11. (Bone): ti, ab, kw (78,375)
  12. (Bony): ti, ab, kw (2,589)
  13. 11 OR 12 (79,571)
  14. (Proximal humerus): ti, ab, kw (534)
  15. (Cement spacer): ti, ab, kw (34)
  16. (Joint replacement): ti, ab, kw (6,213)
  17. (Megaprosthesis): ti, ab, kw (2)
  18. (Endoprosthesis): ti, ab, kw (413)
  19. (Endoprosthetic): ti, ab, kw (95)
  20. (Arthroplasty): ti, ab, kw (17,452)
  21. 16 OR 17 OR 18 OR 19 OR 20 (19,590)
  22. 10 AND 13 AND 14 AND 15 (0)
  23. 22 AND 21 (0)

## Appendix B. Risk of bias (ROB) assessment tools used for the study

**Table:**

1.	**A clearly stated aim**: The question addressed should be precise and relevant in the light of available literature.
2.	**Inclusion of consecutive patients:** All patients potentially fit for inclusion (satisfying the criteria for inclusion) have been included in the study during the study period (no exclusion or details about the reasons for exclusion).
3.	**Prospective collection of data:** Data were collected according to a protocol established before the beginning of the study.
4.	**Endpoints appropriate to the aim of the study:** Unambiguous explanation of the criteria used to evaluate the main outcome which should be in accordance with the question addressed by the study. Also, the endpoints should be assessed on an intention-to-treat basis.
5.	**Unbiased assessment of the study endpoints:** Blind evaluation of the objective endpoints and double-blind evaluation of the subjective endpoints. Otherwise, the reasons for not blinding should be stated.
6.	**Follow-up period appropriate to the aim of the study**: The follow-up should be sufficiently long to allow the assessment of the main endpoints and possible adverse events.
7.	**Loss to follow-up less than 5%:** All patients should be included in the follow-up. Otherwise, the proportion lost to follow up should not exceed the proportion experiencing the major endpoint.
8.	**Prospective calculation of the study size:** Information of the size of detectable difference of interest with a calculation of 95% confidence interval, according to the expected incidence of the outcome event, and information about the level for statistical significance and estimates of power when comparing the outcomes.
*ADDITIONAL CRITERIA IN CASE OF COMPARATIVE STUDY:*	
9.	**An adequate control group:** Having a gold standard diagnostic test or therapeutic intervention recognized as the optimal intervention according to the available published data
10.	**Contemporary groups:** Control and studied group should be managed during the same time period (no historical comparison)
11.	**Baseline equivalence of groups:** The groups should be similar regarding the criteria other than the studied endpoints. Absence of confounding factors that could bias the interpretation of the results
12.	**Adequate statistical analyses:** Whether the statistics were in accordance with the type of study with calculation of confidence intervals or relative risk

The revised and validated methodological index for non-randomized studies (MINORS) questionnaire

## Appendix C.

**Table:**

Study (Author/year)	MINORS risk of bias assessment tool questionnaire												Total Score^*^
	1	2	3	4	5	6^¶^	7	8	9	10	11	12	
Manfrini et al (2011)	2	2	0	2	0	2	2	0	2	2	2	2	18
Kundu et al (2013)	2	2	0	2	0	2	2	0	NA	NA	NA	NA	10
Rafalla et al (2017)	2	2	0	2	0	2	2	0	2	2	2	2	18
Guo et al (2019)	2	2	0	2	1	NA^#^	0	0	NA	NA	NA	NA	7
Gulia et al (2021)	2	2	0	2	1	2	2	0	NA	NA	NA	NA	11
Ebeid et al (2022)	2	2	2	2	0	2	2	0	2	2	2	2	20
Sehrawat et al (2024)	2	2	0	2	0	2	0	0	2	2	2	2	16
Khan et al (2024)	2	2	0	2	1	2	0	0	NA	NA	NA	NA	9

NA – Not applicable ¶ Stipulated average follow up was kept at 2 years post completion of treatment. # Patients with expected survival time > 12 months were excluded from the study ***Scoring criteria:** Not reported = 0; Reported but inadequate = 1; Reported and adequate = 2. Total scores are summed as follows: -Comparative studies: Maximum = 24; Minimum = 0 (≥ 19: Good; 14-18: Fair; ≤ 13: Poor) -Non-comparative studies: Maximum = 16; Minimum = 0 (≥ 13: Good; 9-12: Fair; ≤ 8: Poor)
